# Autonomous 3D Exploration of Large Structures Using an UAV Equipped with a 2D LIDAR

**DOI:** 10.3390/s19224849

**Published:** 2019-11-08

**Authors:** Margarida Faria, António Sérgio Ferreira, Héctor Pérez-Leon, Ivan Maza, Antidio Viguria

**Affiliations:** 1 Robotics, Vision and Control Group, University of Seville, Avda. de los Descubrimientos s/n, 41092 Sevilla, Spain; hectorperez@us.es (H.P.-L.); imaza@us.es (I.M.); 2 Laboratório de Sistemas e Tecnologia Subaquática (LSTS), Department of Electrical and Computer Engineering, School of Engineering (FEUP), University of Porto, 4200-465 Porto, Portugal; asbf@lsts.pt; 3 Center for Advanced Aerospace Technologies, Calle Wilbur y Orville Wright, 19, La Rinconada, 41300 Sevilla, Spain; aviguria@catec.aero

**Keywords:** structure inspection, path planning, unmanned aerial vehicles (UAVs), autonomous exploration, laser scanning

## Abstract

This paper addressed the challenge of exploring large, unknown, and unstructured industrial environments with an unmanned aerial vehicle (UAV). The resulting system combined well-known components and techniques with a new manoeuvre to use a low-cost 2D laser to measure a 3D structure. Our approach combined frontier-based exploration, the Lazy Theta* path planner, and a flyby sampling manoeuvre to create a 3D map of large scenarios. One of the novelties of our system is that all the algorithms relied on the multi-resolution of the octomap for the world representation. We used a Hardware-in-the-Loop (HitL) simulation environment to collect accurate measurements of the capability of the open-source system to run online and on-board the UAV in real-time. Our approach is compared to different reference heuristics under this simulation environment showing better performance in regards to the amount of explored space. With the proposed approach, the UAV is able to explore 93% of the search space under 30 min, generating a path without repetition that adjusts to the occupied space covering indoor locations, irregular structures, and suspended obstacles.

## 1. Introduction

Unmanned aerial vehicles (UAV) are continuously increasing in value as tools and are useful aids for a multitude of human activities. These activities, scenarios, and concepts nowadays cover a wide range of contexts from the multi-vehicle oceanographic environment [1,2,3] to smart farming [4,5], surveillance [6,7,8,9], wildfire tracking [10,11], or transportation [12,13]. These systems have, through miniaturization and reduction in cost, become more attractive and viable especially for tasks which might pose a danger to humans or tasks which are, in their essence, logistical burdens to carry out continuously in a manual manner. The reality is that recent technological developments have opened the use of aerial robots to a broader public with UAVs now benefiting from off-the-shelve long-range wireless communications, high-resolution light sensors, on-board computation capacity, and power-efficient hardware. A prime example of these cumbersome and possibly dangerous tasks is that of mapping unexplored and unstructured areas. In this regard, the capabilities of UAVs are exceptional for scene reconstruction [14,15,16], environmental monitoring [10,17,18], industrial inspection [19,20,21,22], bridge inspection [23,24], and nuclear radiation detection [25] due to the increasing assortment of payloads that these vehicles can accommodate as well as the level of redundancy achievable by UAVs deployed in coordinated teams.

In this work, our goals are two-fold: (1) Enabling the exploration of vast structures with no previous knowledge, and (2) using a low-cost, light laser.

**Figure 1:**
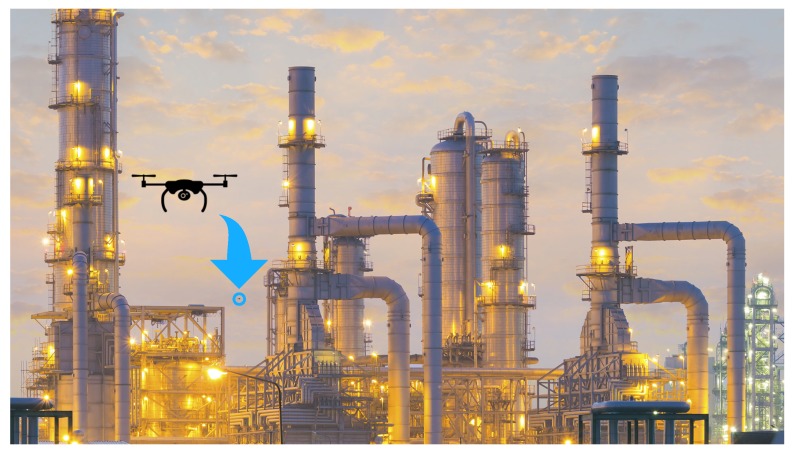
UAV (Unmanned Aerial Vehicle) inspection of large structures.

The simulated operational scenario chosen to experiment the exploration approach is the structural inspection in an industrial setting (see Figure 1). We showcase a system that combines well-known tools with a new sampling manoeuvre to use the potential range of a 2D laser sensor for 3D sampling. The system integrates a conservative path-planner with a layered exploration strategy that considers both the observation manoeuvre and a safety distance, thus leveraging both the operational objectives and the platform’s safety. These distances and the overall size of the search space are addressed by clearly distinguishing between local space (within sensor range) and global space (the full search space). The sampling locations are discovered by searching for frontiers, voxels in the known free space that have unknown neighbours. The frontiers’ algorithm is only the first step towards selecting a goal, with neighbouring unknown points targeted for inspection only when an inspection manoeuvre is possible and safe for the platform to perform. After the system identifies the locations with information gain, the sampling manoeuvre is adjusted based on the map configuration and the sensor range. The path planning time is reduced by adjusting the maximum search time to the scope of the exploration (local or global) and by only selecting goals that have a high likelihood of being reachable. The presented system is an extensible solution, applicable to other environments and operational scenarios beyond exploration.

A significant takeaway of our work is the bridging of the gap between academic state-of-the-art equipment and tools currently available to industry. Considering that industrial structures can reach considerable dimension and magnitude, the ability to employ a systematic and autonomous solution which produces results within a reasonable time opens the door to lower operational costs. However, the generation of a flight plan that yields full coverage of a complex infrastructure remains challenging as sensor characteristics, UAV velocity, and error sources must be jointly taken into account to avoid multiple flights. UAV inspection as a service, has already achieved mainstream status and the value of incorporating our autonomous 3D inspections is clear, avoiding the escalation of current UAV service costs.

The proposed approach articulates well-known components to achieve online and onboard, fully autonomous exploration of large 3D heterogeneous structures without prior information. The main contributions are (1) using a 2D laser for 3D exploration to reduce both cost and payload weight; (2) presenting a sampling manoeuvre that consolidates the world representation while providing sampling flexibility; and (3) the analysis of the system based on experiments done using hardware-in-the-loop simulations in flight-ready components.

The remainder of this paper is organised as follows. Section 2 provides an overview of related work. Section 3 describes the proposed approach for the exploration system. Section 4 details the software components of the system and how they interact. Section 5 describes the methodology used for testing and data collection. Section 6 presents the simulation results collected and Section 7 closes the paper with the conclusions and future lines of research.

## 2. Related Work

The problem of exploration is well known in research. The theoretical exercise of considering a robot a geometrical point has been progressively adapted to the reality of guiding a UAV as it maps a 3D, unknown world.

The exploration strategies proposed in [26] have been repeatedly adapted to UAVs, solving also path planning with the Rapid Exploring Random Trees (RRT) algorithm. Bircher et al. [19] proposes an architecture where RRT plans online with a receding horizon, sampling possible positions. It is an example of modular architecture, where the choice of the objective function switches planning for either the exploration of unknown volume or inspection of a given surface. Papachristos et al. [20] uses RRT to tackle the added challenge of exploring a degraded visual (dark) GPS–denied environment. The RRT is part of a two-step, receding horizon, belief space-based approach that first generates branches to maximise information gain and in a second step, a new tree is sampled to minimise localisation uncertainty, generating collision-free paths. Song and Jo [27] present a system for high-resolution 3D reconstruction combining both the volumetric map and the reconstructed surfaces to evaluate the model completeness. Space is divided into visiting sectors, taking advantage of the spatial organisation of the octree that is given a visitation order with A*. The algorithm first extracts inaccurately reconstructed surfaces by analysing the quality and trend of the surfaces from the Truncated Signed Distance Fields (TSDFs) and extracts frontiers from the volumetric map. Frontiers, introduced by Yamauchi [28], are defined as known, free space that has neighbouring unknown space. RRT is used to select a goal within a sector, maximizing the unknown visible space and penalizing distance. Focusing on the speed of the UAV, Cieslewski et al. [29] takes advantage of the camera’s field of view to restrain the search space in a way that enables the UAV to sustain the speed. The classical frontier algorithm is responsible for selecting the goal, first searching inside the field of view, and then, when there are no frontiers inside the frustum of the camera, searching globally. Again, sampling-based RRT* is responsible for generating the path. Witting et al. [30] also used the field of view to restrict the search space coupled with a RRT path planner. Instead of frontier finding, the goals are selected using a historically aware Next Best View Planner (NBV), adjusting the orientation of the UAV to maximise information gain.

Other approaches do not use sampling based algorithms for path planning, as is the case in Heng et al. [14]. This work solves both the exploration and the coverage problems with a two-step approach. Firstly, the goal state is chosen through maximization of the information gain, weighted by the cost to get there. Then, a path to the goal is generated maximizing coverage, with the given path cost and planning time budgets. To cross a high-density scenario, such as a forest, Oleynikova et al. [31] use a methodology similar to the NBV problem. Sampling from within the view of the sensor, multiple intermediate goals are selected to maximise a goal-seeking reward and exploration gain. Charrow et al. [16] achieves active mapping with a two-stage approach of local and global planning. As a first step, several paths are calculated using Dijkstra’s algorithm with the motion primitives to reach frontiers clustered into regions of high information gain. The selected trajectory is then refined using a gradient-based approach by maximizing an information-theoretic objective function based on Cauchy–Schwarz quadratic mutual information.

However, the stochastic nature of the sampling-based approaches makes the certification for industrial use more complex. Wang et al. [32] employs Information Potential Fields (IPF) for planning. The IPF contains both frontiers as interest regions and the obstacles as repelling fields. The exploration strategy is history aware to avoid local minima. Yoder and Scherer [24] uses the concept of a surface frontier, a free voxel with both an unknown and an occupied neighbour. Surface frontiers are favoured by the utility function to complete the surface inspection without exploring the entire scenario. In this approach, the user defines regions of interest that contain only one connected surface volume. To generate the path in a deterministic way, the path planner SPARTAN creates a sparsely connected graph across a tangential surface around obstacles maintaining a minimal clearance.

Juliá et al. [33] presents a comparative study of exploration strategies both for a single vehicle and for cooperative exploration. This study compares the results of the nearest frontier, the utility function maximization by González-Banos and Latombe [26], and a behaviour-based approach by Lau [34] for single-vehicle exploration. The nearest frontier is expanded to multiple vehicles inspired by the work of Burgard et al. [35]. Similarly, the utility function is expanded to multi-vehicle exploration using a market-based approach, as presented by Zlot et al. [36]. Additionally, two systems that integrate the localization uncertainty are included in the study. One system uses a utility-cost function from the work of Makarenko et al. [37] and a hybrid approach by Juliá et al. [38] that uses behaviour to return to positions with low uncertainty. This study establishes the application’s goal as the decisive factor to choose the exploration strategy. When a high map quality is sought, the integrated techniques achieve better results. If minimizing the exploration time is the decisive factor, cost-utility must be addressed, knowing that utility techniques have a high information acquisition rate at first but increase the total exploration time because small parts of the environment are only explored at the end. In multi-robot approaches, when the number of robots increases, both map quality and execution time improve. However, global optimisation is best if each robot chooses the target, as is the case in market-based approaches.

When the objective of the UAV is to collect surface information, exploration must address the NBV problem in order to place the sensor favourably. Delmerico et al. [39] compares the impact of the volumetric information method on the surface coverage for NBV for occlusion aware, unobserved voxel, rear side voxel, rear ride entropy, proximity count, area factor, and average entropy. Concluding that the reconstruction can achieve most of its model completion within less than ten well-chosen views, regardless of the choice of volumetric information formulation.

Regarding the sensor adopted to sample the world, for exploration in two dimensions, 2D laser sensors have been extensively used. For instance, Rekleitis et al. [40] applies it in planetary exploration and Kaufman et al. [41] equips an UAV with a laser for the purpose of generating a 3D map although the exploration is conducted in 2D. However, the most common sensor used for exploration with UAVs are depth cameras [14,16,19,20,27,29,32,39]. There are also examples of using a 3D LIDAR, combined with depth cameras [24].

Another aspect that gains relevance in an industrial setting is the minimum distance to an obstacle. In the related work presented here, when declared, the minimum distance to an obstacle varies from none in [14,16], to 0.6 m in [29], to 2 m in [24]. Additionally, some approaches optimistically consider the unknown space as free [39].

In this paper, we propose a deterministic, autonomous system for 3D exploration using a 2D laser sensor. The system is able to run online and on-board an UAV that guarantees a minimum distance to obstacles of 2.5 m, while conservatively considering the unknown space as an obstacle. The developed modular architecture applies the frontier algorithm both at local and global levels, expanding the concept of a frontier surface into a surface neighbourhood. Safe paths are generated during the mission by the Lazy Theta* algorithm which is an any-angle variation of the popular A* algorithm.

## 3. Methods

The proposed approach brings together well-known components and a few novel ones to enable autonomous exploration taking advantage of the far-reaching measurement range of laser sensors while maintaining a lower cost by using a 2D sensor. In essence, point clouds containing the measurements from the on-board sensor are continuously integrated into a sparse octree, the world representation. All the components share the same representation. The exploration strategy identifies a safe sampling manoeuvre around the most promising target and the path planning algorithm generates a safe path to its start. Finally, the resultant waypoint sequence is merged with the sampling manoeuvre to create a flight path that is then followed by the UAV. The different parts of this approach are detailed in the following sections.

### 3.1. Sparse Occupancy Grid

The measurements taken by the sensor are continuously integrated into a sparse octree using the implementation of the octomap framework [42]. In this data structure, space is discretised in voxels, each one reflecting the probability of containing an obstacle. Occupancy information is provided in the form of three states: (1) Unknown, (2) free, and (3) occupied. The Octomap implementation has a small memory footprint since each node only stores three variables: The maximum occupancy likelihood, a local identifier, and a pointer to the array of children nodes that is only initialised if the voxel contains known space. It is a sparse world representation because when voxels with the same state are together, they merge into a larger voxel. The variable resolution is the primary characteristic that allows the system to scale to large scenarios. Larger voxels allow significant amounts of space to be analysed at the same time while using smaller voxels to represent intricate details.

### 3.2. Sampling: Flyby Manoeuvre

Laser sensors have the advantage of longer sampling range when compared with cameras and depth-cameras. However they are heavier and more expensive. Fortunately, 2D lasers and lighter and cheaper than their 3D counterpart.

An observation manoeuvre has been developed to enable the 2D laser sensor to capture information in 3D. Instead of sampling from only one viewpoint, sampling is performed along a line segment. In this way, the circular range of the sensor is extended in the direction of the movement. Additionally, the manoeuvre adds flexibility to the thin scan of the 2D sensor. The procedure promotes the consolidation of the known space by smoothing the edges of the unknown space, enabling the voxels to merge, making the world representation more compact.

**Figure 2:**
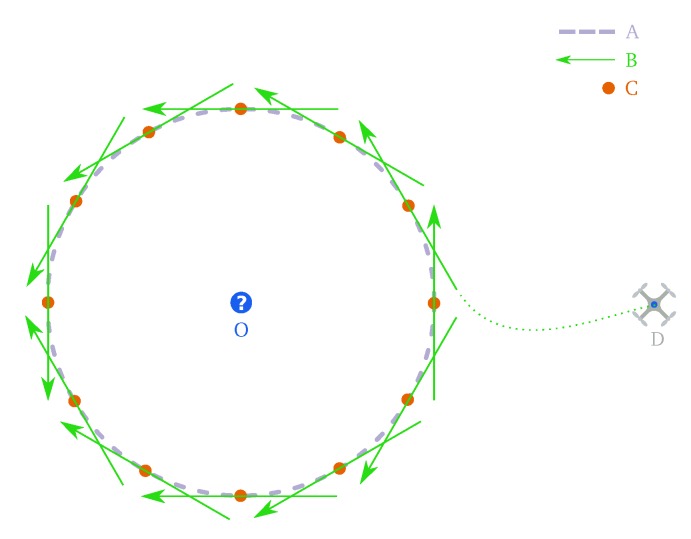
Circle (A) around the unknown location (O) with a radius equal to the sampling distance where different observation points (C) are shown. The green arrows represent the corresponding flyby manoeuvres for each observation point. The UAV is initially at position (D) and the final flyby manoeuvre is chosen to have the shortest path between this position and the start location of the flyby segment.

Then, the manoeuvre consists on following a segment chosen to place the sensor within the range of the targeted unknown space. As shown in Figure 2, the line segment computation can be adapted to the current shape of the free space since several possibilities around the target are evaluated. Each line is a tangent to a circle centered on the target, with a radius equal to the sampling distance. Both the sampling distance and the number of flyby options evaluated are adjustable. However, it should be noticed that more flyby choices increases both flexibility and computation time.

### 3.3. Path Planning

The path planner selected is the Lazy Theta* any-angle deterministic planner proposed in [43]. Its implementation was validated for large structures in outdoors experimental campaigns in previous work of the authors [44]. The sparse resolution of the octomap allows it to avoid large obstacles in paths with up to 90 times the resolution of the occupancy grid. The geometrical optimization for obstacle detection enables a safe flight corridor up to 10 times the resolution of the octree. The obstacle avoidance capabilities are thus able to support operational safety distances of several meters from both obstacles and unknown space. Due to the any-angle characteristics of Lazy Theta*, the path is smooth enough to be used directly by the path follower.

### 3.4. Frontier Algorithm

**Figure 3:**
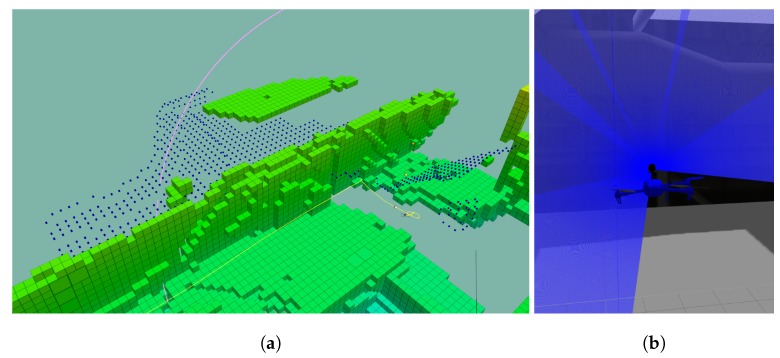
Neighbour generation, according to the sensor range. (**a**) The frontiers in blue could not be sampled as the only known space is above and the laser scanner has a blind spot below due to the 270° scan angle. (**b**) The blue rays illustrate the range of the sensor. The scan angle is pointed upwards.

The classical and widely-used frontier exploration algorithm presented in [28] is adopted to identify information gain. The frontier voxels favour the conditions to move conservatively (as they are in known space) while increasing the information about the world. The frontier algorithm also relies on a sparse representation of the world (the octomap). As a result, the variable size of the frontier and its neighbours creates a spatial organisation of the world beliefs that allow larger segments of space to be analysed in one iteration. The implementation used is an extension of [45], that generates neighbours taking the sensor range into account. Figure 3a shows in blue discarded frontiers because the orientation of the scan angle illustrated in Figure 3b. As the sensor has a 270° scan angle and is mounted to have the blind angle facing the ground at 30°, the information acquisition must be made either at the same height of the unknown space or below it.

Instead of just identifying frontiers, the unknown neighbours that qualify a voxel as a frontier are the candidates for sampling. The implicit spatial organisation of the octree, with its 8 children, is preserved to order the sampling candidates without further calculations.

### 3.5. Exploration

Operational requirements, such as safety distance, observation manoeuvre visibility, or obstacle detection, are combined with the exploration optimisation, such as switching between local and global search, and the circular iteration of the tree on a global search. Furthermore, the maximum amount of time allowed for path planning changes according to the scope of the exploration. The local search has up to $\frac{1}{8}$ of the time allocated in a global search. The iteration of the map between global searches is circular. One global search starts where the last global search finished. This iteration distributes the focus among the whole tree, avoiding analysing the same first voxels more frequently.

The resulting path is combined with the flyby waypoints and the current position. The UAV executes the flyby manoeuvre twice, which affords sampling redundancy: First, the UAV has the sensor facing the movement and then the sensor is facing backwards.

## 4. System Architecture

**Figure 4:**
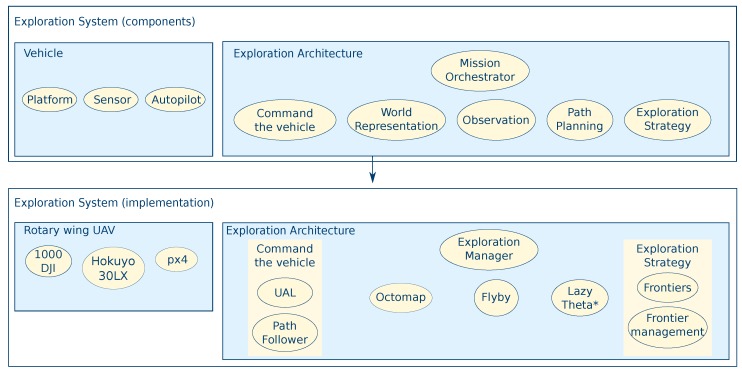
System architecture showing the correspondence between the generic elements and the chosen components and algorithms.

The system architecture detailed in Figure 4 is divided into two large areas: Vehicle and Exploration Architecture. As shown, these areas are abstractions that can be easily interchanged for different types of systems as long as the sub-components, which will be detailed throughout this section, follow the same architecture.

The software architecture is based upon the Robot Operating System (ROS) software framework by Quigley et al. [46], which allows a hardware-agnostic design through the use of its interprocess communication interfaces. The different components are described in the following:

- **Sensor**: The sensor is a Hokuyo 30LX 2D laser sensor with a range of 30 m;
- **Software for basic commands execution**:
  - *UAL (UAV Abstraction Layer):* A software-interface for hardware abstraction [47] which handles the standard commands to control the vehicle such as velocity control, taking-off, and landing;
  - *Path Follower:* Software to follow a waypoint sequence [48], while also adjusting vehicle yaw, so that in every segment the Hokuyo sensor is aligned with the movement.
- **Octomap**: Occupancy octree for world representation using the octomap framework [42], as previously detailed in Section 3.1. The world representation is shared among all the components;
- **Flyby manoeuvre**: The manoeuvre executed to collect data around the target to gather 3D information with the 2D laser, as described in Section 3.2;
- **Path Planning**: The Lazy Theta* any-angle deterministic planner proposed by [43] and adapted in previous work of the authors [44] due to the advantages mentioned in Section 3.3;
- **Exploration Strategy**:
  - *Frontier Algorithm*: The classical and widely-used frontier exploration algorithm presented in [28]; The implementation used is an extension of [49], that generates neighbours taking the sensor range into account as presented in Section 3.4;
  - *Frontier Management*: Combines and orders the operational requirements, such as safety distance, observation manoeuvre visibility, or obstacle detection, with exploration optimisation. The characteristics presented in Section 3.5 are incorporated in this component.
- **Exploration Manager**: Orchestrates all other components to achieve the high-level mission goal of the whole-scenario exploration.

To select a suitable unknown point to sample, the frontiers algorithm searches inside a boundary and identifies several candidate frontiers, listing their unknown neighbours by order of proximity. Afterwards, the Frontier Management component searches for a safe flyby manoeuvre around each unknown point with only the caveat that manoeuvres selected: (1) If there is visibility between the flyby and the unknown point; (2) if the flight corridor of the flyby is within free space; and (3) if the start of the flyby is reachable by the path planner. Furthermore, this component first searches locally, around the current position of the UAV when no frontiers are found and then escalates to the operator-defined region (global search). At a global level, the iterator of the tree is circular and the search within the octree continues after the last successful search to avoid analysing the same voxels repeatedly. Once a flyby is selected, the path planner generates a sequence of waypoints to reach the start of the next flyby. After the UAV reaches the endpoint of the flyby, the search for frontiers starts again. Finally, when no more frontiers are found, the Exploration Manager component declares the mission as finished.

### 4.1. Exploration Manager

The Exploration Manager is an event-driven ROS node with the highest level of abstraction. This node is the linchpin of our implementation and serves to integrate all other components. Within this component, the flow of data is directed from Path Planning to the Exploration Strategy and then to the Path Follower nodes. This module is responsible for deciding whether to continue collecting data or to declare the exploration finished.

### 4.2. Frontier Algorithm

The Frontier Algorithm is used by request, as a service of a ROS node. Each call expects the boundaries of the search region, the desired amount of sampling points, and the option to continue a previous search. The points are returned in a list ordered according to the frontier heuristic.

#### Frontier Manager

The Frontier Manager node combines the information gain targets found by the Frontier Algorithm node and the sampling manoeuvre possibilities to adjust the flyby to the map configuration. Several mechanisms are embedded to reduce the time spent selecting the next goal of the UAV.

**Figure 5:**
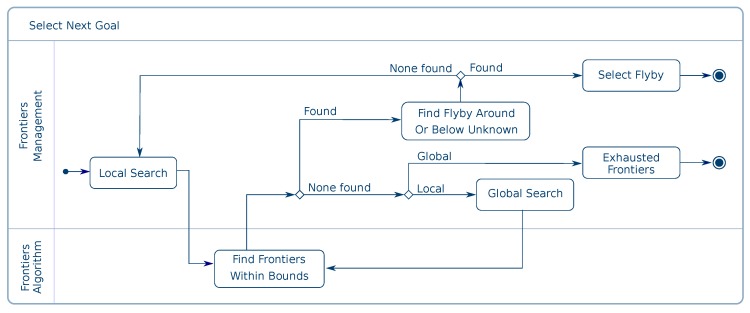
The activity diagram of the Frontier Manager node to find the next goal.

Figure 5 contains an activity diagram of the decision-making process. The starting point is the local search. The local search is centered on the last successful flyby manoeuvre. The boundaries of the local region are always within the operator defined region while keeping a minimum volume. The Frontier Algorithm’s node is requested to compile an ordered list of unknown points. If no unknown space exists, the Frontier Algorithm node is queried again, this time using the operator-defined boundaries (global search). Additionally, the analysis of the map in each global searches is circular as one global search starts where the last global search finished. The focus of the search is distributed among the whole tree, avoiding analysing one set of voxels more frequently.

A flyby manoeuvre will only be considered valid for flying when three requirements are met. These requirements are ordered to minimise the number of line of sight checks. Firstly, the unknown point must be visible from the flyby, assuming the unknown space to be free. Secondly, the minimum distance from obstacles must also be observed when the UAV is sampling. The flight corridor of the flyby must only contain free space to be valid. Finally, the voxel that contains the start of the flyby must have at least one neighbour eligible to be part of the solution of the path planner. Since the octree is sparse, the flight corridor between the start and end of the flyby and the flight corridor between the start of the flyby and its neighbours can be substantially different. This requirement exempts the system from making impossible path requests. Moreover, a reduced amount of flyby options is considered for each sampling point. In order to keep the computation time per sampling candidate compatible with online, on-board execution only nine flyby options are considered for each with six options around the candidate and three below it. Flyby options above the unknown are not considered because the blind angle of the Hokuyo sensor points downwards. The relative position of the manoeuvre is calculated, taking into account the sensor pitch.

### 4.3. Operator Interaction

The system requires minimal input from the operator, only the limits of the inspection region are required, along with starting the UAV within an obstacle-free zone. A starting zone of at least 8 × 8 × 3 m provides a safe operational baseline to execute a small predefined flight plan, which is the starting manoeuvre. At its start location, with the initial manoeuvre, the UAV creates a bubble of known-safe space that will be incrementally expanded through exploration. Since the UAV only navigates within known free space, the starting manoeuvre boot-starts the generation of the map to enable the first autonomous manoeuvres. Consequently, the initial obstacle-free zone is a requirement to operate with the proposed approach. The mission ends either when this bubble completely encompasses the limits defined by the operator or when there are no more avenues to expand the known space (due to obstacles).

With the exploration finished, the resulting output can be used in three broad ways. (1) Immediately, an operator can see a rough reconstruction of the environment. (2) The flight plan generated by the system can be logged and reused in following inspections to gather further measurements, either with the same platform or with another of similar configuration. (3) Finally, all the point clouds generated by the sensor during the flight, cross-referenced with location and time tags, can be processed with off-the-shelf 3D reconstruction software to generate a highly detailed 3D reconstruction of the structure. This last step feature is beyond the scope of this paper.

### 4.4. Modular Approach and Re-Usability

As observed by Delmerico et al. [39], the orthogonality of each of the tasks involved in the system inspires a modular architecture that allows a fast reconfiguration of software for different applications and robotic set-ups.

**Figure 6:**
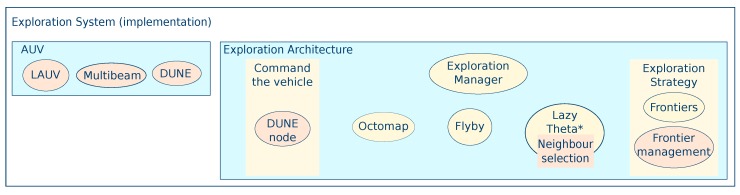
The architecture applied to the case of an AUV (Autonomous Underwater Vehicle). The changes are highlighted in orange and are primarily at the hardware level.

Although this architecture caters to a rotary-wing holonomic UAV, non-holonomic UAVs, and autonomous underwater vehicles (AUVs) can run the system on-board just as readily. The most significant change lies within the hardware, which can be adapted in a plug and play fashion. To exemplify, Figure 6 shows how the components would change for an AUV adopting the LSTS (Laboratório de Sistemas e Tecnologia Subaquática) toolchain [50]. The vehicle becomes a Light AUV (LAUV) equipped with a Multibeam and running on-board DUNE (Uniform Navigational Environment). Additionally, the same Upboard processor used in the UAV field experiments described in [44] is supported by OceanScan’s LAUV to run the exploration system on-board. As DUNE already provides path following for the LAUV, the component to command the vehicle would only need to interface the standard commands from ROS.

The considerations for a non-holonomic vehicle are confined to two components: The waypoint selection within the path planner and the operational requirements embedded into the frontier management.

## 5. Simulation Testbed

In order to validate our assertions and results, a simulation testbed was created to benchmark the system. This testbed provides a Hardware-in-the-Loop (HitL) simulation environment to collect accurate measurements of the capability of the system to run online and on-board.

This work focuses on the task of exploration. The complex problem of localisation is assumed to be addressed with a differential GNSS in outdoor environments and a VICON/OptiTrack system in indoor scenarios. Consequently, in simulations, the localisation used to construct the map is sourced directly from the simulator.

All the code developed to implement the proposed approach is open-source and available online (https://github.com/margaridaCF/FlyingOctomap_code).

### 5.1. Comparison with State of the Art Approaches

**Figure 7:**
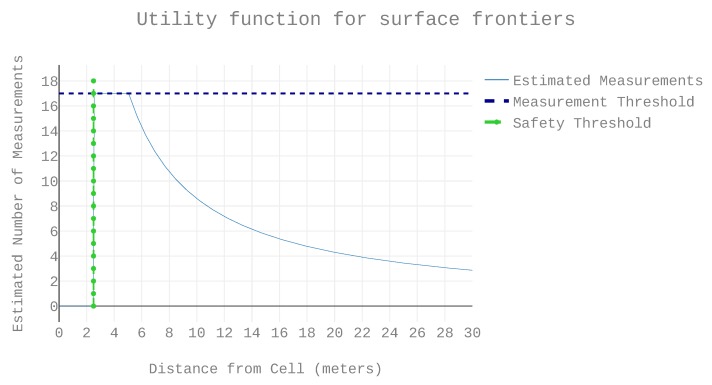
Equation (1) with h=0.5 m, N=1080, $d_{s}=2.5$ m, and $t_{m}=17$.

The frontier heuristic proposed in this work (referenced as octomap heuristic) is compared with two other heuristics from the state of the art. One is the classical nearest neighbour heuristic, originally introduced in [28]. The other is the heuristic presented in [24], where priority is given to occupied space. In [24], only frontiers with occupied neighbours are considered for sampling, and those frontiers are referenced as surface frontiers. As the work in [24] relies on a combination of sensors that allows an assumption of omnidirectional sensing and in this work the sensor is 2D, the utility equation was adapted from 3D to 2D for a fair comparison. The result is Equation (1), where the safe flying distance from the structure is $d_{s}$, and $t_{m}$ is a maximum desired measurements per cell. Given the cell height *h*, the distance from the sensor to the cell *r*, and the number of points per scan *N*, the utility of a view for observing a single surface frontier is

(1) $$f\left(r\right)=\left\{\begin{array}{cc} 0, & \mathrm{if}\;r<d_{s}\\ t_{m}, & \mathrm{if}\;r⩽r<d_{m}\\ \frac{hN}{2\pi r}, & \mathrm{if}\;d_{m}⩽r \end{array}\right.$$

where $d_{m}=\frac{hN}{2\pi r}$. The domain of Equation (1) is equivalent to the utility function used in [24]. The function is plotted in Figure 7 using the reference vehicle’s sensors parameters and thresholds.

The redundancy in the flyby manoeuvre (flying forwards and backwards) and allowing the frontier to be at any point in the map is considered part of the proposed approach. Consequently, when employing the surface frontier and the nearest neighbour heuristics, the flyby manoeuvre is done only in one direction and the frontiers must be at a distance shorter than 45 m, the distance the Lazy Theta* implementation is designed to reach in the allotted time.

### 5.2. Hardware in the Loop

**Figure 8:**
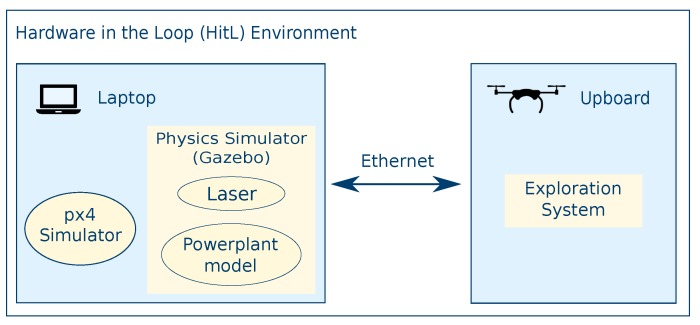
The diagram of the Hardware-in-the-Loop (HitL) environment details how the load balancing between the Upboard and the support laptop reflect the resources available in a real flight.

To employ a simulation environment that is valid in an experimental setup, the vehicle used in an outdoors experimental campaign in previous work [44] is taken as a reference to collect the data. The same on-board processor provides the computational power to run the ROS nodes of the exploration system. An additional computer simulates the environment, emulates the sensor, and simulates the autopilot (see Figure 8). The details are as follows:

1. **Platform**: A 1000 DJI frame with sufficient payload to mount all the necessary hardware: A 5.8 GHz wireless communication Ubiquiti® Rocket, the autopilot, the on-board processor, and the sensor;
2. **On-board processor** An UpBoard with an Intel^®^ Atom^TM^x5;
3. **Sensor**: The sensor is a Hokuyo 30LX laser sensor with an aperture of 270° and a range if 30 m, mounted with a 50° pitch;
4. **Autopilot**: The Pixhawk v1’s autopilot px4 provides software-in-the-loop capabilities that simulate the vehicle’s movements during the tests;
5. **Support laptop**: A OMEN HP-15-ce020ns equipped with an Intel^®^ Core^TM^ i7-7700HQ.

### 5.3. Test Setup

**Figure 9:**
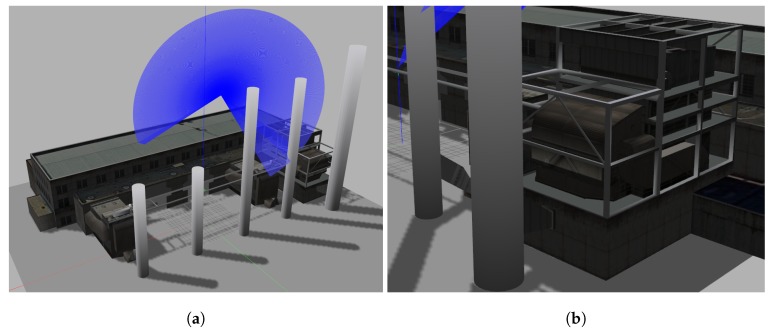
The model used to simulate the inspection scenario. (**a**) The factory to inspect with the UAV and a rendering of the sensor range. (**b**) A part of the structure impossible to cover safely.

The 3D scenario constructed inside Gazebo contains the model of a power plant obtained from the Gazebo model library (http://models.gazebosim.org/), as illustrated in Figure 9a. The operator sets a subset of the model as the region of interest. The UAV starts the flight between the pillars and the building. This region includes various types of obstacles: Two thin rails suspended in mid-air, a beam structure, and an indoor portion. Not all parts of the structure can be accessed because of the minimum distance to obstacles, as shown in Figure 9b.

The parameters used in this setup are specified in Table 1.

#### Metrics

The behaviour of the system will be analysed over ten runs. In each run, data is collected to evaluate the following metrics:

1. The exploration time;
2. The volume explored;
3. The resulting map contextualised with the flight path;
4. The path length of the flight path;
5. The evolution of occupied space during the mission;
6. The time spent in path planning;
7. The rate of success of path planner;
8. The average execution time per view;
9. Entropy of the map in the final iteration.

The first eight aspects are the most commonly used among the following works [19,20,27,29,31,39,51,52,53,54,55,56].

Entropy [57] is also analysed to provide an insight about the information gain when the exploration finishes. The entropy $H_{i}$ of the *i*-th voxel with occupancy probability $p_{i}$ is computed using Equation (2).

(2) $$H_{i}=-\left(p_{i}\log p_{i}+\left(1-p_{i}\right)\log\left(1-p_{i}\right)\right)$$

## 6. Results and Discussion

The system was run ten times in the HitL environment to gather performance information according to the previous metrics and this section describes the results.

### 6.1. Execution Time

To understand the bottlenecks of the system, the execution time of the mission manager was broken down into tasks. From the view of the mission manager, the time of the mission was divided into one of three tasks: Visiting waypoints, planning the path, or analysing the known world to find the next sample point.

**Figure 10:**
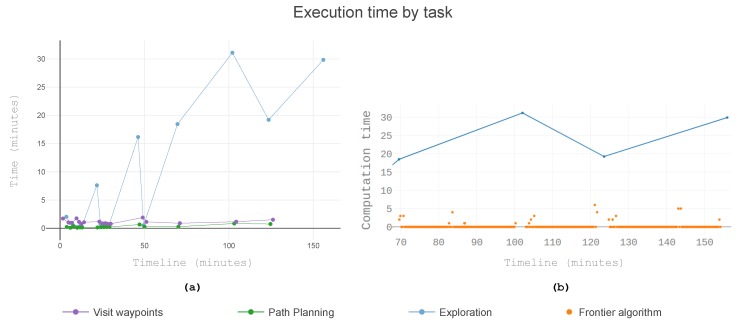
Time used in exploration in one run with a linear iterator. (**a**) Elapsed time for each task. (**b**) The total time for the exploration task and the time used in the frontiers algorithm.

A preliminary study showed exploration to be the longest task to execute. The data is illustrated in Figure 10. In this graph, the task of exploration had an uneven execution time. The task could complete faster when restricting the search space to local exploration. Oppositely it could take longer when all the space is analysed (global exploration). Due to the impact on the overall execution time, the global search was optimised. As a first approach, the map is always searched from the same starting point. Figure 10b shows a detail containing the last four global searches as well as the execution time of the frontier algorithm. Each search was fast, but an increasing number of searches were needed. Moreover, for each frontier, all the safety checks were performed. For each potential goal, up to nine flyby options were analysed. For each flyby, space was discretised to search for unknown space and obstacles, along with all the required checks. In short, for each flyby, the goal manager went through each requirement of safety.

**Figure 11:**
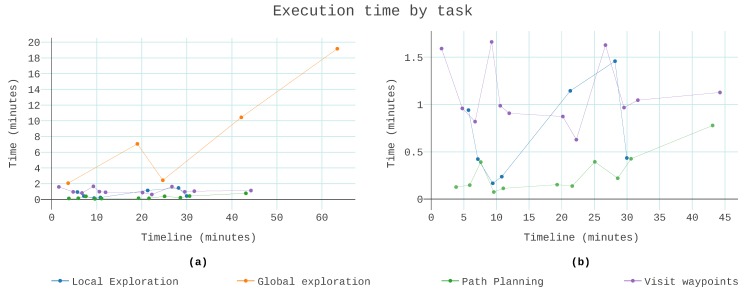
The execution time the mission manager spent in each task, in one run with a circular iterator. (**a**) All four tasked scaled together and (**b**) just local exploration, path planning, and visit waypoints.

The critical insight of Figure 10b is that fewer frontier searches resulted in a shorter task time. The off the shelf iterator of octomap started the search always at the same point. As the mission progressed, it became less likely that the first frontiers found would be suitable goals. If these voxels were still frontiers, it was because there were obstacles in their vicinity that prevented safe sampling. A circular iterator was introduced to disperse the focus of the search through the whole tree. With this iterator, the search began where the last global search selected a goal. One voxel was only considered again after examining the whole tree. The circular search ensured that the known space has significantly changed when next considering that particular voxel. The impact is shown in Figure 11. There were only two instances of exploration taking over 8 min, as opposed to the six occurrences seen in Figure 10a. Additionally, the maximum exploration time dropped from 30 min to 19 min.

Figure 11a shows all tasks together. Except for global exploration, none of the tasks took more than 2 min. By adjusting the maximum search time of the planner, it resulted in a success rate of 84% to find a path. The bottleneck of the system was the global exploration. The last global search was the one that took more time. It served only to verify that the exploration had finished, the collection of data ended with the last waypoint visited. Figure 11b is a detail of the same dataset. The global exploration was removed. The last waypoint visited roughly 45 min, which was 19 min sooner than the end of the exploration.

**Figure 12:**
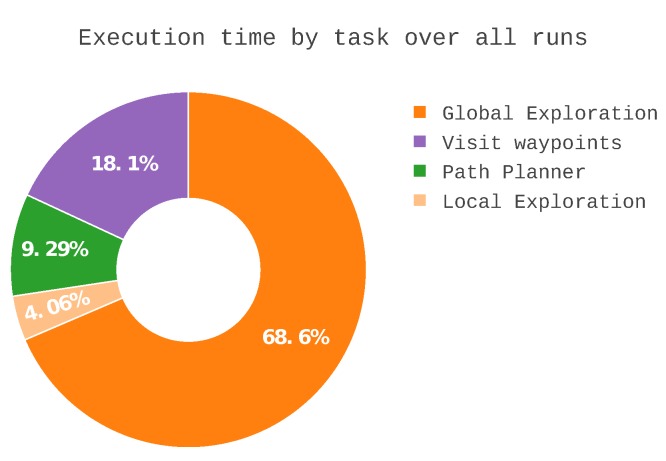
Distribution of the execution time of each task across ten runs.

When this analysis extended to all the runs, as shown in Figure 12, the global exploration was consistently responsible for over half the total mission time. In terms of computation time per view, the average was 6 min and 38 s ± 1 min and 56 s. The median was 6 min, bounded by 5 min and 19 s in the best case and 10 min and 28 s in the worst case. Due to the limited options to sample the unknown space, the frontiers were often not observable. Although the resulting processing time per view was high, the location variability increased as more voxels were analysed, affording a higher information acquisition.

**Figure 13:**
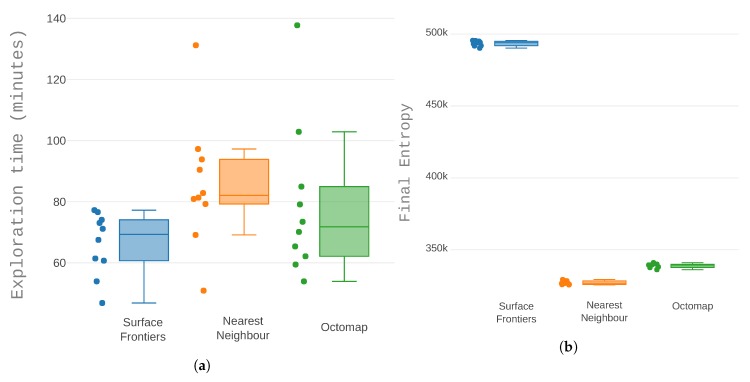
A comparison of the exploration with different heuristics: Nearest neighbour, octomap sector order, and closest surface frontier. Each heuristic was run 10 times. (**a**) Distribution of the time the system needs to complete the exploration task and (**b**) total entropy at the end of each run.

In terms of the total time to complete an exploration sequence, on average, the system took 78 min ± 30 min. Figure 13 shows a large difference in total execution time: The shortest run (run 3) only took 59 min and with greater coverage of occupied space, whereas the slowest run (run 5) took more than double the amount of time, 2 h and 18 min.

However, as seen in Figure 13a, the surface frontiers heuristic and the octomap heuristic had similar execution times, on average. The first finished in under 1 h and 20 min whereas the second used 72 min and the other 69 min. The similarity is not expected “a priori”, considering that surface frontier heuristic did not visit the segments of the scenario with only free space. However, there were repeated occurrences of much longer execution time with the octomap heuristic and with the surface frontiers heuristic there were none. The nearest neighbour had longer execution times. With an average run time of 1 h and 22 min and a maximum run that reached 2 h and 11 min. The heuristic determined not only the average run time but also the bounds of what run times that could be expected.

In terms of entropy, the differences are clear. The surface heuristic had a drastically higher entropy at the end of the exploration. This can be explained as it was focused on occupied space and frontiers without occupied space in their vicinity were not even considered for sampling. The other two heuristics had a lower entropy which was expected because both accepted frontiers around free space as sampling targets. The nearest neighbour heuristic achieved the best entropy results with a small difference compared to the octomap approach.

### 6.2. Volume Explored

**Figure 14:**
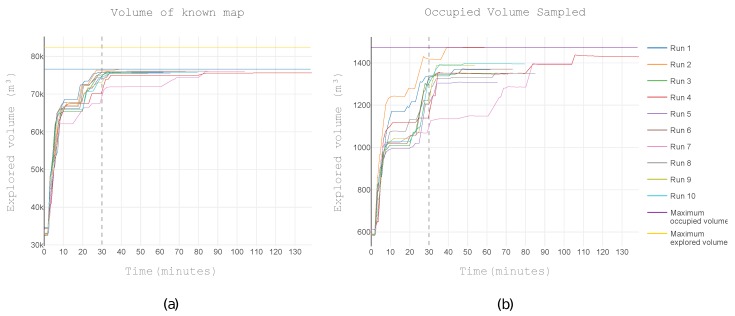
The volume explored in 10 runs. (**a**) Rate of exploration: Free and occupied are combined. (**b**) Coverage of the occupied volume.

Another important metric is the volume explored. Figure 14a shows the progression of known space in 10 runs. The highest rate of information acquisition happened within the first half-hour highlighting the effectiveness in making the reconnaissance of the scenario.

Figure 14b shows the rate of discovery of occupied space where the first half-hour also contained the highest discovery rate. This space state was the most relevant for structural inspection. Run 2 achieved both the highest map completeness (93%) and the highest surface coverage. All the components of the exploration architecture were deterministic, yet there was variability. The fluctuation came from the movement of the UAV. In each run, the UAV occupied slightly different positions. This change was enough to create different map configurations. Part of the space was unreachable due to obstacles. Within the reachable space, most of the space was free. Of the reachable space, the exploration covered an average of 92% ± 0.4%.

**Figure 15:**
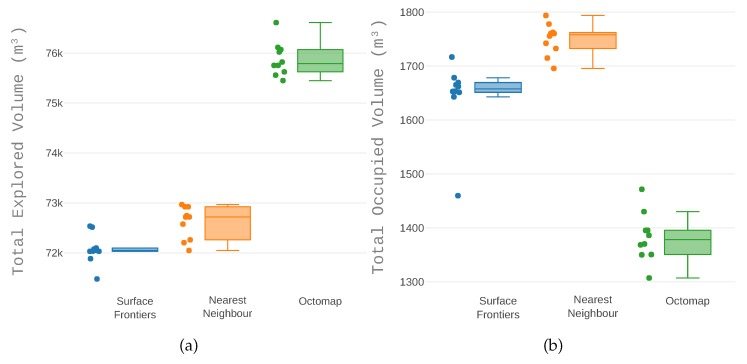
The volume explored at the end of the run for each of the analysed heuristics. Both (**a)** the total explored volume and (**b**) the volume of occupied space. The scales of the explored volume is one order of magnitude higher than the amount of occupied space, consequently (**b**) magnifies the difference of the outcome of each heuristic.

The volume explored at the end of the run for each of the analysed heuristics is shown in Figure 15. The octomap heuristic explored significantly more space, 3.071 m${}^{3}$ (4%) more space than the nearest neighbour heuristic and 3.749 m${}^{3}$ (5%) more space than the surface frontiers heuristic.

The nearest neighbour and the surface frontiers heuristics achieved similar results both for the total explored space and for the occupied space. Regarding explored volume, the octomap heuristic stood out for exploring more space in total (the sum of free and occupied). When only analysing occupied space, the octomap heuristic discovered less space. However, the difference was one order of magnitude more significant for total space than it was for occupied space.

### 6.3. Flight Path

**Figure 16:**
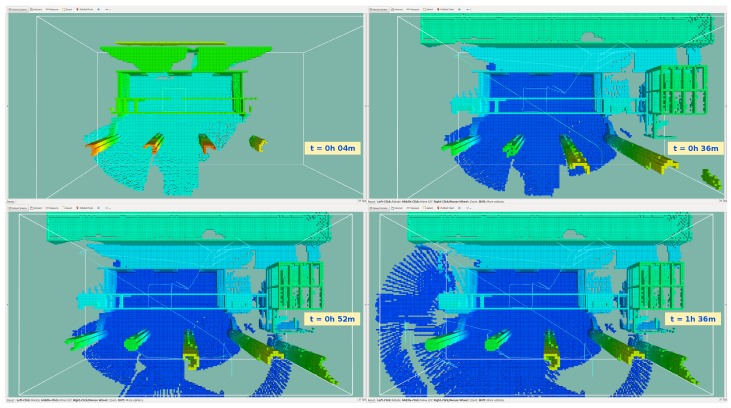
The evolution of occupied space present, throughout a mission cycle. A snapshot is taken at the end of the initial manoeuvre and every 30 min after that. The last snapshot shows the final map. The light blue line illustrates the path taken by the vehicle. The white outline represents the search space defined by the operator.

To illustrate the progression of the structure exploration throughout a run, Figure 16 shows the evolution of the occupied space. A snapshot was taken at the end of the initial manoeuvre and every 30 min after that. The first two snapshots show the most significant change in occupied space.

**Figure 17:**
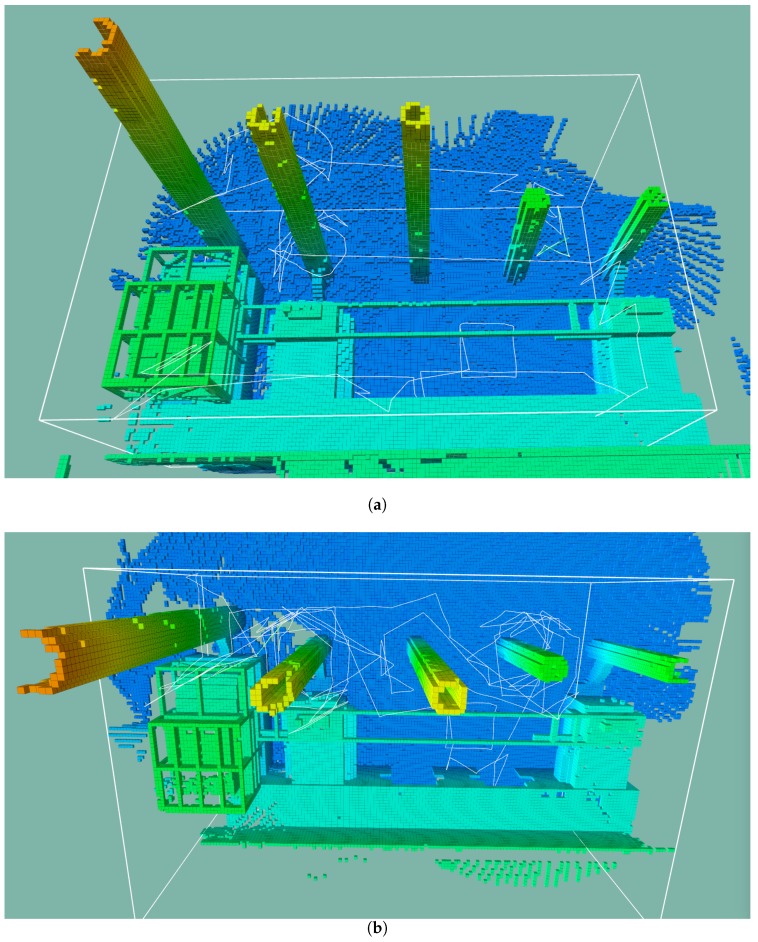
A comparison of the final map with different heuristics. The path is represented as a white line. The search space is represented as a white box. The measurements are incorporated into the world representation even if they are outside the navigational bounds defined by the operator. (**a**) Nearest neighbour and (**b**) surface frontier.

By comparing Figure 16d with Figure 17a,b the characteristics of each heuristic are visible. The nearest neighbour and the octomap heuristic resulted in similar paths that sample all the map. The surface frontier heuristic circled the towers and kept to that side of the scenario. With all heuristics, the path entered the open gates.

**Figure 18:**
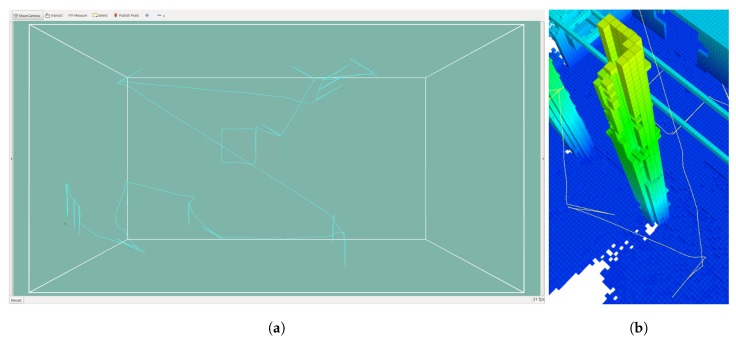
(**a**) Flight path with 507.3 m. (**b**) The flight plan adapts to the features of the structure.

Figure 18a shows the path travelled by the UAV in one of the runs scaled with the map generated by the sensor data collected throughout the path. Each area of the map was visited only once, shortening the length of the flight path. As the flight progressed, the map also progressed towards completion.

**Figure 19:**
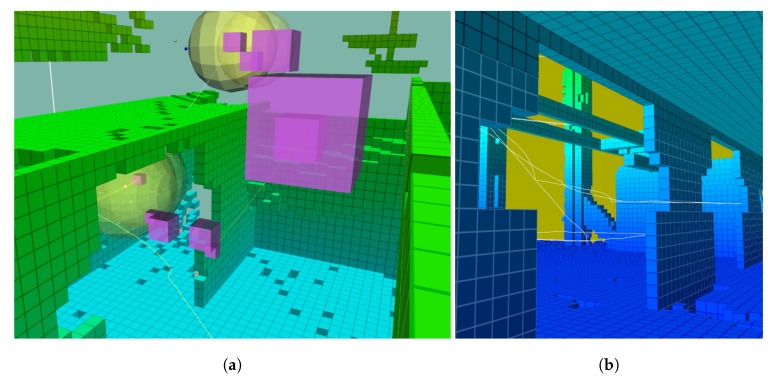
(**a**) Exploration transitions between indoors and outdoors seamlessly. The starting point is below. The target is represented by the unknown point above in dark blue. The yellow spheres represent the safety margin around each point. The required safe flight corridor in each segment has the same diameter. The purple cubes are the voxels containing each waypoint. The different sizes show the sparse quality of the octree. (**b**) The same detail viewed from inside the structure.

The flight path adjusted to the available free space allowing the system to sample heterogeneous shapes. This flexibility allowed for a transition from outdoor to indoor inspection seamlessly, provided that there was enough connecting free space. In Figure 19, the segment of the path started above and outside a building and continued inside. The safety distance was maintained from the walls and the opening. In Figure 19a, there were visible parts of the ceiling that were unknown, but after sampling in (b), the ceiling was fully mapped. The UAV took advantage of the range of the Hokuyo sensor mounted with a 50° pitch to gather information because of the 270° scan angle, which generated a blind angle that faced downwards.

Finally, the path is analysed in the light of the criteria for coverage planning, as defined by Cao et al. [58]:

- The final flight plans could cover an average of 92% of the search space. However, instead of an area, this is now the target volume;
- The region was filled out without overlapping paths;
- The paths were continuous and sequential without any repetition, although its execution was not continuous in time. One exception was made on the observation manoeuvres where the segment had flown both ways to add redundancy of samples;
- The vehicle could avoid all the obstacles, with the added restriction of considering the unknown space as an obstacle;
- Only simple motion trajectories were used, in this case, straight lines;
- The path was not guaranteed to be optimal in length or execution time. However, it achieved the goal of dispensing prior knowledge in less time than it would take the human operator to plan the path and fly the UAV, while also avoiding gaps in the coverage.

## 7. Conclusions and Future Work

This paper presented a deterministic system capable of autonomously inspecting large structures in unknown and heterogeneous scenarios. The resulting system combined well-known components and techniques with a new manoeuvre to use a low-cost 2D laser to measure a 3D structure. This combination allowed the use of the far-reaching laser sensors instead of the more common depth camera. The sampling manoeuvre and the flyby extended the 2D range into 3D.

The resulting flight plan explored 93% of the volume defined by the operator and covered most of the reachable occupied surface in the first 30 min. The effectiveness of conducting the reconnaissance of the scenario suggests having a two-step approach. The first step would be reconnaissance with the proposed approach and a second step to consolidate the measurements. In the second step, each vertex becomes the node of a graph of sampling locations, a Travelling Salesman Problem. The path of the UAV was continuous and without repetition, adapting in three dimensions to the free known space enabling the UAV to cover indoor locations, irregular structures, and suspended obstacles. Without prior knowledge of the world, the system sampled a scenario in a shorter period than the time required for a human operator to plan the path and fly the UAV while avoiding gaps in the coverage. The tests were conducted using a HitL environment identical to the environment used to validate Lazy Theta* in outdoors experimental campaigns in [44].

The exploration strategy successfully identified the locations of information gain with the frontier algorithm, taking advantage of the spatial organisation embedded into the octree to order the frontiers. First, the search for frontiers was done locally, to minimise the length of the flight path, then, if no frontier was available, the global exploration ensured a full coverage of the map. For each frontier, the exploration strategy searched for a flyby sampling manoeuvre that fit into the available free space. Maintaining a forward-facing sensor during the flight led to a maximisation of the information gained.

Using the spatial organisation of the octree, the system was able to explore more than with any of the other heuristics considered in the paper. The heuristic employed to select the frontier is compared with other two approaches from the literature: The classic nearest neighbour approach and an occupied space centric heuristic. The median of the execution time of the three heuristics is similar but the surface frontier has a significantly smaller variability. However, at the end of the exploration, the entropy was markedly higher when using the surface frontier heuristic. The total volume explored reflected the entropy results and the surface frontiers heuristic achieved less known space.

The deterministic property was relevant for industrial tools that require certification to the highest level. The UAV could function as a stand-alone tool since all the calculations were performed online and on-board.

Although the use case in this paper considered a rotary-wing UAV industrial inspection, the system could be applied to other platforms such as AUVs and other applications such as humanitarian relief.

Several avenues of future research are open. A more dynamic approach could adjust the number of flyby orientations according to the obstacles density for applications that need to minimise the unreachable volume. Additionally, generating the flyby by clustering frontiers and finding an orthogonal vector removes the requirement of line-of-sight checks to assess each flyby.

It would be interesting to explore the impact of more significant differentiation between the local and the global exploration strategy through the frontier heuristic, including the occupied neighbours and the nearest distance.

Future works continue towards finding alternative means of maintaining the voxel variability while reducing the exploration execution time.

## Figures and Tables

**Table 1:** Parameters for the HitL data collection.

Parameter	Value	Parameter	Value
$v_{linear}$	1 m/s	$path\_planning\_time_{max\_execution}$	4 min
$v_{angular}$	0.1745 rad/s	local space minimum	10 × 10 × 10 m
Octree resolution	0.5 m	Operator-defined region	70 × 38 × 31 m
$d_{safe}$	2.5 m	Sampling distance	5 m
Frontiers amount	35	Flyby options amount	6
		Flyby length	4 m
